# A prospective observational cohort study: neurotoxic processes associated with psychiatric symptoms in heroin use disorder: the kynurenine pathway and neopterin levels

**DOI:** 10.3389/fpsyt.2026.1785477

**Published:** 2026-04-02

**Authors:** Güleser Akpınar, Mehtap Sarıoğlu, Gözde Girgin, Terken Baydar, Hande Sipahi

**Affiliations:** 1 Department of Emergency Medicine, Yeditepe University, Institute of Health Sciences, Çam ve Sakura City Hospital Programme of Pharmaceutical Toxicology, Istanbul, Türkiye; 2 Çamsakura City Hospital, Istanbul, Türkiye; 3 Department of Psychiatry Bakırköy Mazhar Osman Training and Research Hospital for Mental Health and Neurological Disorders, Istanbul, Türkiye; 4 Department of Toxicology, Faculty of Pharmacy, Hacettepe University, Sihhiye, Ankara, Türkiye; 5 Department of Toxicology, Faculty of Pharmacy, Yeditepe University, Istanbul, Türkiye; 6 Department of Toxicology, Faculty of Pharmacy, University of Health Sciences, Istanbul, Türkiye

**Keywords:** heroin use disorder, kynurenine pathway, neopterin, neuroinflammation, psychiatric symptoms, tryptophan metabolism

## Abstract

**Aim:**

This study investigated the effects of tryptophan (TRP) metabolism, kynurenine pathway (KP) and neopterin levels in heroin use disorder (HUD) in relation to neurotoxicity, treatment processes and psychiatric recovery.

**Method:**

The study groups consisted of patients with HUD who sought treatment at the hospital and received treatment, and a healthy control (C) group. The levels of neopterin, tryptophan and kynurenine in the participants’ serum were determined, and the pre- and post-treatment results in the groups were compared with the controls.

**Results:**

Neopterin levels, reflecting immune system activation, were observed to decrease in HUD group after treatment (p = 0.021). Similarly, a decrease in levels of tumor necrosis factor-alpha (TNF-α), a pro-inflammatory cytokine, was also detected after treatment (p < 0.05). However, changes in interleukin-6 (IL-6) and interferon-gamma (IFN-γ) levels were not found to be significant (p > 0.05). The total scores of the Addiction Profile Index (API-K), which measures the severity of addiction, decreased significantly after treatment (p < 0.001). At the same time, improvement was observed in the general symptom level (GSI) and all subscales (including depression, anxiety, obsessive-compulsive disorders, and somatization) of the Symptom Checklist-90-Revised (SCL-90-R) scale, which assesses general psychopathology. Regression analyses revealed that the two strongest factors negatively affecting treatment success in the HUD group were duration of use (p = 0.028) and high neopterin levels (p = 0.020).

**Conclusion:**

This study highlights the critical role of immunological and neuroinflammatory processes in the success of HUD treatment. The findings indicate that biochemical recovery progresses in parallel with psychiatric recovery during treatment, and that changes in the KYN/TRP ratio and IL-6 levels have strong diagnostic and prognostic value. Neopterin, kynurenine, and tryptophan, on the other hand, have moderate prognostic value.

## Introduction

Heroin, also known as diacetylmorphine, is a potent opioid agonist synthesized from morphine. It rapidly crosses the blood-brain barrier (BBB), exerting a potent effect on the central nervous system (CNS) (1). Chronic heroin use leads to profound neurobiological adaptations that fundamentally alter brain chemistry, structure, and function, resulting in long-term neurotoxicity and behavioral changes (2). Heroin has been shown to suppress immune function by directly interacting with receptors on immune cells. Low levels of pro-inflammatory cytokines are observed in chronic heroin users. However, withdrawal periods and treatment processes can cause the immune system to reactivate and increase the release of pro-inflammatory cytokines (3).

Tryptophan (TRP), in addition to being a precursor to neurotransmitters such as serotonin, is largely metabolized via the kynurenine (KYN) pathway (KP). This pathway produces a series of biologically active metabolites. Some of these metabolites, particularly quinolinic acid (QUIN), may exhibit neurotoxic effects by stimulating N-methyl-D-aspartate (NMDA) receptors, while others, such as kynurenic acid (KYNA), may possess neuroprotective properties (4). Chronic inflammatory conditions disrupt this delicate balance by shifting TRP metabolism away from serotonin production and towards the KP (5). In chronic heroin use, the activation of inflammatory processes leads to the induction of the indoleamine 2, 3-dioxygenase (IDO) enzyme, which causes a shift towards the neurotoxic branch of KP (QUIN). This metabolic shift contributes to both the emergence of depressive symptoms associated with serotonin deficiency and increased neuronal damage due to the neurotoxic effects of QUIN (6).

This represents an important underlying mechanism for the psychiatric symptoms (depression, anxiety) and cognitive impairments observed in heroin use disorder (HUD) (7). Neopterin is a pteridine derivative released by activated macrophages and microglia in response to interferon gamma (IFN-γ) stimulation. It functions as a reliable biomarker of cellular immune activation. Neopterin and IDO activation are both triggered simultaneously by IFN-γ. Therefore, in chronic inflammatory conditions, neopterin increases in biological fluids, including blood, while blood TRP levels decrease, which is considered as an indicator of inflammatory burden (8). In HUD treatment, changes in neopterin levels reflect the immune system’s response to treatment and the resolution of neuroinflammatory processes. Therefore, neopterin levels have both diagnostic and prognostic value (9). Understanding the complex effects of heroin on neurotoxicity is crucial for developing treatment strategies for HUD and preventing heroin-related neurological and psychiatric complications. Therefore, the aim of this study is to investigate neurotoxicity associated with heroin use in the context of its relationship with alterations in TRP–KP metabolic balance, neopterin-mediated immune activation, and psychiatric symptom profile.

## Method

The study was conducted between 1 November 2023 and 1 November 2024 at the Bakırköy Mazhar Osman Mental Health and Neurological Diseases Training and Research Hospital, Heroin and Substance Abuse Treatment and Research Center (AMATEM). The study protocol was reviewed and approved by the relevant Institutional Review Board/Ethics Committee of [Institution] (Approval No: 221909003, Date:30.11.2023). All participants provided written informed consent prior to enrolment, and the study was conducted in accordance with the Declaration of Helsinki.

Inpatients diagnosed with HUD according to The Diagnostic and Statistical Manual of Mental Disorders Fifth Edition (DSM-5) criteria were included in the study. The inclusion criterion for the patient group was individuals aged 18 years and older diagnosed with isolated HUD according to DSM-5 diagnostic criteria (10). Exclusion criteria included the use of steroids, probiotics, antibacterial agents, or nonsteroidal anti-inflammatory drugs within two months prior to the study, abuse of another substance, body mass index (BMI) of 30 kg/m² or above, metabolic disorders such as diabetes, chronic inflammatory diseases, cancer, HIV, neurodegenerative diseases, pregnancy, or neurological disorders.

A total of 61 individuals were included in the study. The study groups consisted of a HUD group of 30 patients and a control (C) group of 31 healthy adults. The C group consisted of healthy volunteers aged 18 years and older with no history of disease and no regular medication use in the past six months. Healthy controls underwent a single fasting morning blood draw at enrolment and were not followed longitudinally.

Blood samples were taken from participants in the morning on an empty stomach (fasting for at least 8 hours beforehand) before the start of treatment and one week after treatment. Biochemical analyses included blood heroin concentrations, alanine aminotransferase (ALT) and aspartate aminotransferase (AST) activities, known hepatitis markers, leukocyte, erythrocyte and platelet counts, haemoglobin, haematocrit, mean cell volume (MCV), vitamin B12 and folate levels were evaluated by the hospital biochemistry laboratory.

Tryptophan and kynurenine analysis was performed simultaneously using high-performance liquid chromatography (HPLC; HP Agilent 1100, Vienna, Austria) (11). The KYN/TRP ratio was used to estimate TRP degradation and was expressed as micromoles of KYN per millimole of TRP.

Measurements of inflammatory markers IL-6, IFN-γ, neopterin, and tumor necrosis factor-alpha (TNF-α) were performed using a commercial enzyme-linked immunosorbent assay (ELISA) method according to the manufacturer’s kit procedures. Human Interleukin 6 (IL-6) BT Lab ELISA Kit (E0090Hu), Human Interferon Gamma (IFN-γ) BT Lab ELISA Kit (E0105Hu), Neopterin IBL ELISA Kit (RE59321), Human Tumor Necrosis Factor α (TNF-α) BT Lab ELISA Kit (E0082Hu) (12).

Psychiatric assessment was performed using the Addiction Profile Index Clinical Form (BAPI-K) and Symptom Checklist-90-Revised (SCL-90-R) scales. The BAPI-K scale measures six different clinical domains and consists of subscales for depression, anxiety, lack of anger C, lack of safe behavior, thrill-seeking behavior, and impulsivity. The SCL-90-R scale consists of 90 questions measuring nine core symptom dimensions and assesses somatization, obsessive-compulsive, interpersonal sensitivity, depression, anxiety, hostility, phobic anxiety, paranoid ideation, and psychoticism (13, 14).

Inpatient treatment and withdrawal management: All HUD participants received standard inpatient care at AMATEM during the withdrawal/treatment phase. Medication exposure, duration of inpatient stay, and relevant clinical characteristics were extracted from clinical records and considered in secondary/adjusted analyses where applicable.

### Statistical analysis

Analyses were performed using SPSS (version 24.0). Continuous variables are presented as mean ± SD for approximately normally distributed data and as median (IQR) otherwise; categorical variables are presented as n (%). Distributional assumptions were evaluated using graphical assessment and normality testing. Independent-group comparisons were performed using the independent-samples t-test (or Welch’s t-test when variance heterogeneity was present) or the Mann–Whitney U test, as appropriate. Within-group pre–post comparisons in the HUD cohort were conducted using the paired t-test or Wilcoxon signed-rank test, as appropriate. Categorical variables were compared using the chi-square test or Fisher’s exact test when expected cell counts were <5. Receiver operating characteristic (ROC) analyses were conducted to evaluate the discriminative performance of biomarker changes (Δ=post-treatment minus pre-treatment) for treatment response, defined as [Treatment response was defined as a reduction of ≥0.5 standard deviations in the BAPI-K total score between baseline and day 7.]. Area under the curve (AUC) values are reported with 95% confidence intervals, and optimal cut-offs were determined using the Youden index with corresponding sensitivity and specificity. Regression analyses were performed to identify variables associated with treatment success, defined as [Treatment success was defined using the same criterion, and logistic regression was used to identify variables associated with treatment success.], using [logistic/linear] regression with reporting of effect estimates and 95% confidence intervals. Two-sided p-values <0.05 were considered statistically significant, and p-values are reported to three decimals (p<0.001 where applicable).

## Results

In this study, the C group (n=31) and individuals with HUD (n=30) were compared in terms of hematological, biochemical, neuroinflammatory, and psychopathological indicators before and after treatment. Changes in the HUD group before and after treatment were evaluated with reference to the C group. The mean age of the HUD group was 34.5 ± 7.6 years, the male ratio was 93.3%, and the high school and below education level was 96.6%. The average age of the C group was 41.5 ± 8, the male ratio was 58.1%, and the high school and lower education level was 6.5%.

The median duration of heroin use was 10 years (6.1–17.8 years). The prevalence of chronic diseases [asthma, hepatitis B (HBV), hepatitis C (HCV)] was found to be 20.0%. Anti-HCV positivity was 13.3%, which was significantly higher than in the C group. Psychiatric medication use was 43.3% (buprenorphine, fluoxetine, hydroxyzine, quetiapine, mirtazapine, naloxone, olanzapine, sertraline, and valproic acid).

Liver function tests revealed that the HUD group had significantly lower enzyme levels compared to the C group. ALT levels were 25.2 ± 15.8 U/L in the C group, while they were 15.3 ± 11.4 U/L at baseline in the HUD group, and this difference was statistically significant (p < 0.01). The increase in ALT levels to 19.2 ± 17.0 U/L after treatment also showed a significant change (p=0.01). Similarly, the AST level was 26.1 ± 8.6 U/L in the C group and 16.8 ± 6.3 U/L at baseline in the HUD group, which was significantly lower (p < 0.01). The increase in AST after treatment was also significant (p < 0.01). When hematological findings were evaluated, WBC was found to be higher in the HUD group compared to the control group (C group 7.2 ± 2.4 ×10³/µL, HUD group at baseline: 9.1 ± 2.4 ×10³/µL), and this difference was significant (p < 0.01). The MCV value was also found to be significantly increased in the HUD group (p < 0.01).

When vitamin levels were examined, vitamin B12 levels prior to treatment were significantly lower in the HUD group compared to the C group (p=0.01). Similarly, folate levels were also found to be lower in the HUD group (p=0.03) (**Table 1**).

**Table 1:** Comparison of Hematological, Hepatic, and Biochemical Parameters Between Healthy Controls (HC) and Individuals with Heroin Use Disorder (HUD).

Parameter (unit)	HC (single measurement)	HUD (Baseline)	p¹	HUD (day 7)	p²
Sociodemographic characteristics					
Age (years)	41.5 ± 8.0	34.5 ± 7.6	<0.001	—	—
Education level, n (%)			<0.001		
Primary school	0 (0)	4 (13.3)		—	—
Secondary school	2 (6.5)	12 (40.0)		—	—
High school	5 (16.1)	13 (43.3)		—	—
University	24 (77.4)	1 (3.3)		—	—
Heroin concentration					
Heroin (ng/mL)	—	≤20.00	NA	ND	NA
Liver enzymes					
ALT (U/L)	25.2 ± 15.8	15.3 ± 11.4	<0.001	19.2 ± 17.0	0.048
AST (U/L)	26.1 ± 8.6	16.8 ± 6.3	<0.001	18.3 ± 6.3	0.067
Hematological parameters					
WBC (×10³/µL)	7.2 ± 2.4	9.1 ± 2.4	<0.001	8.9 ± 2.4	0.548
RBC (×10^6^/µL)	5.1 ± 1.5	4.7 ± 0.4	0.340	4.8 ± 0.4	0.055
PLT (×10³/µL)	262.7 ± 79.3	287.5 ± 91.9	0.030	276.2 ± 84.8	0.247
Hb (g/dL)	13.8 ± 1.7	13.9 ± 1.1	0.840	14.1 ± 1.3	0.084
Hct (%)	43.4 ± 3.7	42.8 ± 3.3	0.600	43.2 ± 3.7	0.346
MCV (fL)	86.4 ± 6.3	91.0 ± 4.3	<0.001	90.5 ± 4.0	0.100
Biochemical parameters					
Vitamin B12 (pg/mL)	374.9 ± 115.0	311.6 ± 102.1	0.010	353.2 ± 92.3	0.001
Folate (ng/mL)	6.44 ± 2.99	5.13 ± 2.79	0.030	5.41 ± 3.04	0.060

Data are presented as mean ± SD for continuous variables and n (%) for categorical variables.

p¹: comparison between HC and HUD baseline (independent samples t-test for continuous variables; chi-square test for categorical variables).

p²: comparison between HUD baseline and day-7 (paired samples t-test).

HC, healthy control; HUD, heroin use disorder; ALT, alanine aminotransferase; AST, aspartate aminotransferase; WBC, white blood cell; RBC, red blood cell; PLT, platelet; Hb, hemoglobin; Hct, hematocrit; MCV, mean corpuscular volume; NA, Not Applicable; ND, None detectable.

In neuroinflammatory biomarkers, TRP levels were significantly lower in the HUD group compared to the C group (p < 0.01), and a significant increase in this level was observed after treatment (p=0.03). The level of kynurenine (KYN) decreased significantly after treatment (p < 0.01). Kynurenic acid was initially higher in the HUD group compared to the C group (p=0.04).

The neopterin level was 6.1 nmol/L in the C group and 5.6 nmol/L in the HUD group, showing a significant difference (p=0.03). The TNF-α level was 83.0 ng/L in the C group and increased to 134.1 ng/L in the HUD group, and this difference was significant (p=0.01). A significant decrease in TNF-α was observed after treatment (p=0.05). Similarly, interleukin-6 (IL-6) levels were 62.0 ng/L in the C group and increased to 114.3 ng/L in the HUD group (p=0.01) and decreased significantly after treatment (p=0.04). IFN-γ levels were also significantly higher in the HUD group compared to the C group (p < 0.01) and showed a significant decrease after treatment (p=0.04) (**Table 2**).

**Table 2:** Comparison of neuroinflammatory biomarkers between healthy controls (HC) and heroin use disorder (HUD) patients at baseline and day 7.

Parameter (unit)	HC (single measurement)	HUD (baseline)	p¹	HUD (day 7)	p²
Neurobiomarkers					
Tryptophan (μmol/L)	68.1 (54.7–73.3)	57.1 (53.2–64.8)	<0.001	59.8 (51.7–72.5)	0.030
Kynurenine (μmol/L)	3.3 (2.5–3.9)	3.3 (2.7–3.6)	0.900	2.6 (2.6–3.3)	<0.001
KYN/TRP ratio (μmol/mmol)	49.8 (42.0–58.0)	51.7 (44.9–59.5)	0.100	47.7 (40.9–59.8)	0.410
Kynurenic acid (nmol/mL)	2.1 (1.5–2.6)	3.0 (2.4–5.6)	0.040	3.5 (1.6–6.6)	0.460
Immune–inflammatory markers					
Neopterin (nmol/L)	6.1 (3.6–9.6)	5.6 (4.5–6.2)	0.030	4.2 (3.9–5.2)	0.800
TNF-α (ng/L)	83.0 (77.5–112.6)	134.1 (67.5–188.0)	0.010	110.6 (80.1–194.4)	0.050
IL-6 (ng/L)	62.0 (2.0–132.3)	114.3 (25.5–166.5)	0.010	113.2 (70.4–174.0)	0.040
IFN-γ (ng/L)	41.2 (36.0–50.1)	63.9 (50.5–91.3)	<0.001	61.5 (41.8–76.7)	0.040

Data are presented as median (interquartile range, IQR).

p¹: comparison between HC and HUD baseline (independent groups; Mann–Whitney U test).

p²: comparison between HUD baseline and day-7 (paired samples; Wilcoxon signed-rank test).

Two-sided p-values are reported to three decimals; p < 0.001 is presented where applicable.

HC, healthy control; HUD, heroin use disorder; TRP, tryptophan; KYN, kynurenine; TNF-α, tumor necrosis factor-alpha; IL-6, interleukin-6; IFN-γ, interferon-gamma.

The total BAPI-K score in the HUD group decreased from 12.8 ± 3.2 before treatment to 10.7 ± 3.0 after treatment (p<0.001). In the subscales, a decrease in anger control (p < 0.05), depression (p=0.01), and anxiety scores was also found to be significant (p=0.04). A notable finding was that the increase in motivation scores showed a significant improvement with treatment (p<0.001) (**Table 3**).

**Table 3:** Changes in BAPI-K Total and Subscale Scores in the Heroin Use Disorder (HUD) Group Before and After Treatment.

Scale	Baseline	Day 7	P
BAPI-K Total Score	12.8 ± 3.2	10.7 ± 3.0	< 0.001
Inadequate Anger Control	3.0 (2.0–3.0)	2.0 (1.0–3.0)	0.045
Lack of Safe Behaviour	5.0 (4.0–6.0)	4.5 (3.0–5.0)	0.067
Thrill-Seeking Behaviour	2.0 (2.0–3.0)	2.0 (1.0–2.5)	0.089
Impulsivity	3.0 (2.0–5.0)	2.5 (2.0–4.0)	0.056
Depression	4.0 (3.0–5.0)	2.5 (2.0–4.0)	0.012
Anxiety	3.0 (2.0–4.0)	2.0 (1.0–3.0)	0.039
Diagnosis	15.0 (13–17)	12.0 (10–14)	< 0.001
Effects on Life	9.2 ± 4.5	6.7 ± 4.1	0.004
Intense Desire	2.2 ± 1.4	1.3 ± 1.0	0.001
Motivation	6.0 (4.0–8.0)	8.0 (6.0–9.0)	< 0.001

Data are presented as mean ± SD for normally distributed variables and median (interquartile range, IQR) otherwise. Within-group comparisons were performed using the paired t-test or Wilcoxon signed-rank test, as appropriate. Two-sided p-values < 0.05 were considered statistically significant.

The SCL-90-R general symptom index decreased from 1.11 ± 0.53 to 0.85 ± 0.57 (p=0.001). Significant decreases were recorded in the somatization, obsessive-compulsive symptoms, depression, anxiety, phobic anxiety, paranoid ideation, and psychoticism subscales (p<0.01). Compared to the C group, the GSI difference before treatment was 1.01 (p<0.001), while after treatment it decreased to 0.6 (p<0.001) (**Table 4**).

**Table 4:** Changes in SCL-90-R total and subscale scores in the HUD group before and after treatment, with baseline comparison to healthy controls.

Scale	HC (baseline)	HUD (baseline)	HUD (day 7)	p
General Symptom Index (GSI)	0.96 ± 0.47	1.11 ± 0.53	0.85 ± 0.57	0.001
Somatisation	0.32 ± 0.37	0.75 ± 0.64	0.47 ± 0.53	0.001
Obsessive–Compulsive	0.53 ± 0.46	1.11 ± 0.79	0.78 ± 0.69	0.001
Interpersonal Sensitivity	0.32 (0.1–0.6)	1.18 (0.7–1.8)	0.89 (0.5–1.4)	0.003
Depression	0.50 ± 0.50	1.34 ± 0.85	0.96 ± 0.79	0.001
Anxiety	0.28 ± 0.37	1.10 ± 0.78	0.75 ± 0.70	< 0.001
Anger/Hostility	0.17 (0.0–0.5)	1.03 (0.5–1.7)	0.67 (0.3–1.3)	0.001
Phobic Anxiety	0.14 (0.0–0.3)	0.64 (0.3–0.9)	0.21 (0.1–0.5)	< 0.001
Paranoid Ideation	0.67 ± 0.50	1.25 ± 0.72	0.96 ± 0.68	0.003
Psychoticism	0.20 (0.0–0.5)	0.58 (0.2–0.9)	0.30 (0.1–0.7)	< 0.001

Data are presented as mean ± SD for normally distributed variables and median (interquartile range, IQR) otherwise. Within-group comparisons (HUD baseline vs day-7) were performed using the paired t-test or Wilcoxon signed-rank test, as appropriate. Between-group comparisons at baseline (HC vs HUD) were conducted using the independent samples t-test or Mann–Whitney U test. Two-sided p-values < 0.05 were considered statistically significant.

**Figure 1:**
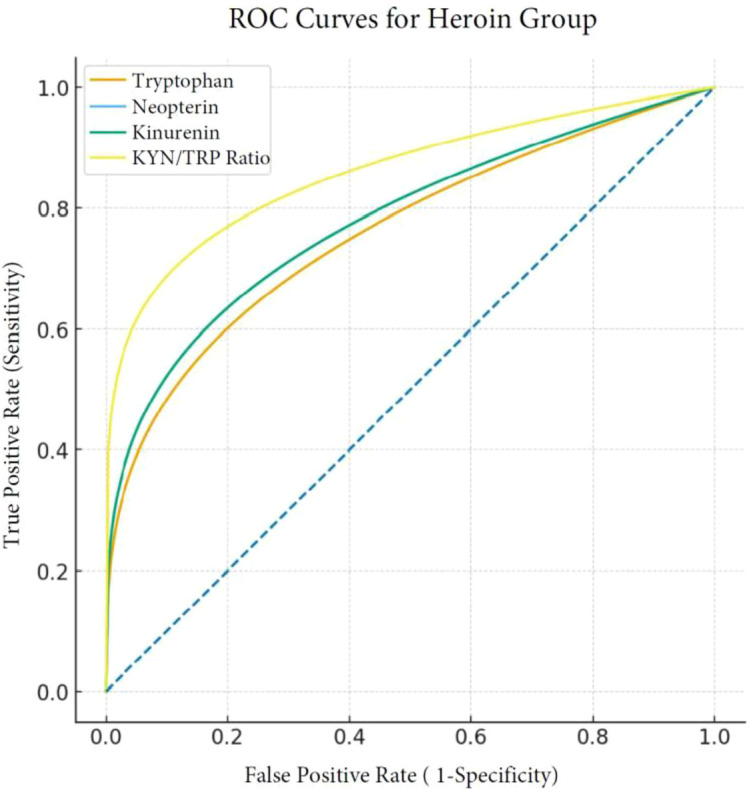
Predictive power of HUD treatment response according to changes in neuroinflammatory biomarkers.

In ROC analyses, the change in the KYN/TRP ratio had the highest predictive value (AUC = 0.86, sensitivity=80%, specificity=82%). The change in IL-6 levels also showed high predictive value (AUC = 0.81, sensitivity=78%, specificity=77%). The AUC values for neopterin, kynurenine, and tryptophan ranged from 0.76 to 0.78 (**Table 5**, **Figure 1**).

**Table 5:** Predictive performance of changes in neuroinflammatory biomarkers for treatment response in the HUD group.

Biomarker (Δ)	AUC (95% CI)	Cut-off	Sensitivity % (95% CI)	Specificity % (95% CI)
Neopterin	0.78 (0.61–0.95)	≤ -0.9	70 (42–85)	78 (55–93)
Tryptophan	0.76 (0.59–0.93)	≥ +4.0	60 (36–80)	67 (42–85)
Kynurenine	0.78 (0.61–0.95)	≤ -0.25	68 (42–85)	76 (48–89)
KYN/TRP ratio	0.86 (0.72–1.00)	≤ -0.8%	80 (55–93)	82 (55–93)
TNF-α	0.68 (0.49–0.87)	≤ -0.7	60 (36–80)	65 (42–85)
IL-6	0.81 (0.65–0.97)	≤ -1.2	78 (55–93)	77 (55–93)
IFN-γ	0.75 (0.57–0.93)	≤ -0.4	73 (48–89)	75 (48–89)

Δ denotes the change from baseline to day-7 (post-treatment minus pre-treatment). ROC analysis was performed to evaluate prediction of treatment response. Optimal cut-off values were determined using the Youden index. AUC 95% confidence intervals were assuming responder and non-responder distribution (n_1_=6, n_0_=24). Treatment response was defined as a reduction of ≥0.5 standard deviations in BAPI-K total score between baseline and day-7. Sensitivity and specificity confidence intervals were calculated using the Wilson method.

## Discussion

This study analyzed changes in pre- and post-treatment levels of markers such as the KYN pathway, neopterin, and proinflammatory cytokines in individuals with HUD. These findings indicate that treatment for HUD involves a complex recovery process encompassing not only behavioral changes but also profound biological alterations. The younger age group of patients may be partially explained by the early onset of substance use and the rapid development of addiction. Differences in educational level reveal the socio-economic dimension of addiction. The fact that individuals with a higher educational level respond better to treatment suggests that psycho-educational and cognitive approaches may increase the effectiveness of treatment programs (15, 16).

The marked increase in B12 and folate levels after treatment in the HUD group demonstrates the positive effect of the treatment process on nutritional status. This increase in vitamins indicates that nutritional deficiencies commonly seen in HUD are being remedied with treatment and that the body’s metabolic balance is being restored (17). The increase in ALT observed in the HUD group may be related to the prevalence of hepatitis C infection in this group, but it may also reflect the temporary liver effects of the drugs used in the treatment process and structural repair due to regular nutrition (18).

Neopterin is a marker secreted by macrophages and monocytes, particularly those stimulated by IFN-γ, and reflects the activation of the cellular immune system (8). The decrease in neopterin levels with treatment (p = 0.02) indicates that the immune activation caused or sustained by chronic heroin use is suppressed. This finding emphasizes that addiction is not only a neurotransmitter imbalance but also a chronic inflammatory condition, and that treatments targeting this inflammatory process should be considered in addiction treatment. TNF-α may contribute to increased cytokine release and increased glutamate release by enhancing microglia activation, which can lead to neurotoxicity (19). The reduction in TNF-α levels with treatment may contribute to the suppression of this neurotoxic cascade and the preservation of neuronal health in the central nervous system. In our study, a decrease in TNF-α levels was observed after treatment (p < 0.05). This indicates that the balance of these pro-inflammatory cytokines is restored with treatment. This change demonstrates that addiction treatment also has positive effects on immune system functions. IL-6 is a pleiotropic cytokine with both pro-inflammatory and anti-inflammatory properties and can play different roles under different conditions (20). IFN-γ, on the other hand, is the main regulator of the Th1 type immune response and plays a critical role in IDO activation. In our study, changes in IL-6 and IFN-γ levels were not found to be statistically significant. The KYN/TRP ratio is accepted as an indicator of IDO activity. A decrease in the KYN/TRP ratio with treatment indicates a return to normalization of the patient’s biochemical profile, i.e., as inflammation decreases, IDO activity decreases and more TRP is directed towards serotonin synthesis (6). This can reduce the neurotoxic load and explains the improvement observed in patients’ mood at the biochemical level. Excessive accumulation of KYNA may also inhibit α7 nicotinic receptors and contribute to impairments in cognitive processes (21). Thus, the imbalance in KP may lead to excitotoxicity due to excess QUIN on the one hand, and cognitive dysfunction due to excess KYNA on the other. Inflammatory conditions induce kynurenine monooxygenase enzyme, increasing QUIN production, while in anti-inflammatory conditions, KYNA production may increase. The net effect of this balance on the nervous system depends on the severity and duration of inflammation. Additionally, it has been reported that chronic heroin exposure and the accompanying inflammatory processes may accelerate biological aging, and that these changes in the immune system may not fully return to normal even during weeks of withdrawal. This highlights the long-term nature of neuro-immuno-endocrine dysregulation in addiction and suggests that environmental factors such as early life stress may further complicate the recovery process of the immune system and neuronal health (22, 23).

The clinical manifestations of immunological recovery are quite significant. Significant decreases in BAPI-K and SCL-90-R scores demonstrate that biochemical improvement parallels psychiatric recovery. The significant decrease in total BAPI-K scores (p <0.001) indicates that individuals experienced significant improvements in their daily living activities and social functioning. This improvement occurred not only as a result of cessation of substance use but also due to the comprehensive treatment approach (24). The decrease in the SCL-90-R Global Symptom Index (GSI) and improvements across all subscales indicate that the treatment is effective across a broad spectrum of psychopathology. In particular, significant improvements in depression, anxiety, and obsessive-compulsive symptoms highlight the close relationship between these symptoms and addiction and the positive effect of treatment on these comorbid conditions (25). In the HUD group, duration of use (p = 0.03) was found to be one of the strongest negative predictors. Long-term substance exposure is thought to cause more persistent and difficult-to-reverse changes in the brain and immune system. The adverse effect of chronic heroin use on neuroplasticity and treatment response indicates the need to develop preventive approaches and early diagnosis-treatment strategies (26). The fact that high baseline neopterin levels are one of the strongest predictors of treatment failure (p = 0.02) suggests that patients with a higher neuroinflammatory burden at the start of treatment have a lower potential for recovery. This finding suggests that neopterin may not only be a marker of condition but also have prognostic value. Monitoring neopterin levels throughout treatment and considering additional anti-inflammatory approaches in patients who do not show a decrease may be reasonable (3).

A significant indirect effect of the change in the KYN/TRP ratio on the reduction in addiction severity (ΔBAPI-K) was found. From a mechanistic perspective, a decrease in the KYN/TRP ratio leads to an improvement in the serotonergic system and a reduction in the effects of KYN metabolites in the brain. Possible consequences of this are increased stress tolerance, reduced impulsivity, and stabilization of mood swings (26).

## Conclusion

The KYN/TRP ratio demonstrated the highest discriminatory capacity among the evaluated biomarkers in assessing neurotoxic burden. The high diagnostic success of neopterin and IL-6 levels suggests that inflammatory markers may play an important role in assessing neurotoxicity associated with addiction. Assessing these parameters at the start of treatment may help identify patients at high risk of neurotoxicity and facilitate the development of individualized, neuroprotective treatment plans. Thus, managing HUD becomes possible by targeting not only the addictive behavior but also the underlying neurotoxic processes.

### Limitations

Several limitations of this study should be acknowledged. First, the sample size was relatively small, particularly in the subgroup analyses, which may limit the statistical power and generalizability of our findings. The HUD group consisted predominantly of males (93.3%) and individuals with low education level (96.6%), and the findings may not be fully representative of female patients or those with different sociodemographic backgrounds. Second, although we evaluated the HUD group before and after treatment, the control group was assessed only at a single time point. This limits our ability to assess spontaneous changes over time in healthy individuals and to make causal inferences about the observed changes in the HUD group. Third, the treatment period and its content were not standardized across all participants. The HUD group received various psychiatric medications (e.g., buprenorphine, antidepressants, antipsychotics), and the duration of treatment varied. These differences may have influenced the biochemical, neuroinflammatory, and psychopathological outcomes independently of the effect of heroin use cessation. Fourth, potential confounding factors such as nutritional status, smoking, alcohol use, and other substance use were not fully controlled. Low vitamin B12 and folate levels observed in the HUD group may be related to dietary deficiencies rather than direct effects of heroin use, and these factors may also influence neuroinflammatory markers. Fifth, the presence of chronic viral infections (HCV, HBV) in the HUD group may have affected inflammatory markers such as neopterin, TNF-α, and IL-6. Although these conditions were documented, their individual effects were not analyzed separately due to the small sample size. Sixth, this study relied on self-report scales for psychopathological assessment, which may be subject to social desirability and recall biases. Additionally, the BAPI-K and SCL-90-R, although validated instruments, may not capture the full complexity of psychiatric symptoms in this population. Finally, the cross-sectional comparison with the control group and the pre-post design without a long-term follow-up period limit our ability to evaluate the sustainability of the observed improvements. Longitudinal studies with longer follow-up are needed to determine whether the changes in neuroinflammatory markers and psychopathological symptoms persist over time.

## Data Availability

The raw data supporting the conclusions of this article will be made available by the authors, without undue reservation.
